# Narrowing Perceptual Sensitivity to the Native Language in Infancy: Exogenous Influences on Developmental Timing 

**DOI:** 10.3390/bs3010120

**Published:** 2013-02-06

**Authors:** Mayada Elsabbagh, Annette Hohenberger, Ruth Campos, Jo Van Herwegen, Josette Serres, Scania de Schonen, Gisa Aschersleben, Annette Karmiloff-Smith

**Affiliations:** 1 Centre for Brain and Cognitive Development, Birkbeck, University of London, WC1E 7JL, UK; E-Mails: ruth.campos@uam.es (R.C.); j.vanherwegen@kingston.ac.uk (J.V.H.); a.karmiloff-smith@bbk.ac.uk (A.K.); 2 Department of Psychiatry, McGill University, 1033 Pine Avenue West, Montreal, H3A 1A1, Canada; 3 Department of Cognitive Science, Middle East Technical University, METU, Ankara, 06800, Turkey; E-Mail: hohenberger@ii.metu.edu.tr; 4 Laboratoire Psychologie de la Perception, University Descartes-CNRS, Paris, 75006, France; E-Mails: josette.serres@parisdescartes.fr (J.S.); scania.de-schonen@parisdescartes.fr (S.S.); 5 Department of Psychology, Saarland University, Saarbrücken, D-66123, Germany; E-Mail: aschersleben@mx.uni-saarland.de

**Keywords:** speech processing, infancy, mother-infant interaction, contingency

## Abstract

The infancy literature situates the perceptual narrowing of speech sounds at around 10 months of age, but little is known about the mechanisms that influence individual differences in this developmental milestone. We hypothesized that such differences might in part be explained by characteristics of mother-child interaction. Infant sensitivity to syllables from their native tongue was compared longitudinally to sensitivity to non-native phonemes, at 6 months and again at 10 months. We replicated previous findings that at the group level, both 6- and 10- month-olds were able to discriminate contrasts in their native language, but only 6-month-olds succeeded in discriminating contrasts in the non-native language. However, when discrimination was assessed for separate groups on the basis of mother-child interaction—a ‘high contingency group’ and a ‘moderate contingency’ group—the vast majority of infants in both groups showed the expected developmental pattern by 10 months, but only infants in the ‘high contingency’ group showed early specialization for their native phonemes by failing to discriminate non-native contrasts at 6-months. The findings suggest that the quality of mother-child interaction is one of the exogenous factors influencing the timing of infant specialization for speech processing.

## 1. Introduction

Within the first few years of life, infants acquire their native language with remarkable ease. Not only do neonates differentiate their own language from languages belonging to other language families [1], but they also prefer listening to their mother’s voice and poems/stories she read aloud before they were born (see review in [2]). Debate continues as to whether some components of speech and language are genetically determined [3,4], but there is little controversy about the fact that infants show remarkable readiness and sensitivity to acquiring language and that experience contributes to shaping these abilities over development. Recent advances in developmental science have paved the way for elucidating how initial endogenous biases interact with language exposure and other exogenous factors to allow infants to tune into the sounds and structures of their mother tongue.

Over the first year of life, infants’ perceptual capacities become progressively more specialized in a fashion consistent with the rhythmic and prosodic patterns of their mother tongue [5,6,7]. At 6 months, infants are able to discriminate speech sounds from a variety of different language families. However, by 10–12 months of age, their perceptual abilities are narrowed to those sounds specifically relevant to their own language [8,9,10]. The nature of the decline in perceptual sensitivity is tightly bound to the characteristics of the language being acquired, implying that the perceptual system does not simply turn on or off a particular speech contrast, but rather that the system undergoes substantial dynamic reorganization during this early period [8,11]. 

Exactly how infants make their gradual journey from an open system comprising a wide variety of sounds to the specialized system observed in adults is a complex, interactive developmental story, the elements of which are gradually beginning to be understood. At the most basic level, neural and perceptual systems enable the infant to discriminate among human speech sounds [12]. Language exposure, combined with the statistical learning capacities that infants possess [13], play an important role in sensitizing the infant to the frequency and distributional properties of the exposure language [14,15], resulting in an impact on syllable structure and phonotactics [16]. The timing and consistency of exposure appear to be highly relevant for the acquisition of speech contrasts. For instance, Korean adoptees who were no longer exposed to Korean after 3–8 years of age were no better at discriminating Korean contrasts compared to monolingual French speakers [17]. On the other hand, infants exposed to a language early on who receive continuous exposure, even for just a few hours per week alongside their native language, do show native-like discrimination skills [18]. 

A further influence on the development of speech in infancy is the social context in which language acquisition takes place. Interestingly, ‘live’ social interactions were found to be crucial to the acquisition of speech because exposure to the same contrasts through television does not result in learning [19]. Specifically, research has demonstrated that infants are particularly sensitive to patterns of contingency in the context of social interactions. For example, infants given contingent phonological feedback from their mothers will rapidly restructure their babbling, incorporating phonological patterns from caregivers’ speech, whereas infants who are provided the same feedback in a non-contingent fashion will not [20]. This suggests that contingency in social interaction plays an important role in acquisition of speech milestones. 

The goal of our study was to map individual differences in reaching speech discrimination milestones onto differences in the quality of mother-child interaction, and in particular onto contingency in those interactions. To test the relationship between contingency in the context of naturalistic mother-child interactions and speech discrimination, we focus on a well-replicated milestone where infants narrow their perceptual sensitivity to native language contrasts. Little research has examined the mechanisms involved and how qualitative differences in mother-child interaction might relate to this ability. Infant sensitivity to syllables from their native tongue was compared to sensitivity to non-native phonemes (from a different language family) in a discrimination task. The study had two aims: (1) to replicate the finding of loss of discrimination of non-native phonemes between 6- and 10-months of age in English, as well as extending the findings to two new languages, French and German, and (2) to explore the relationship between the narrowing of this phonological sensitivity and contingency of mother-child interaction. We chose the 6–10 month time window of longitudinal change to assess the impact of exogenous factors, such as mother-child interaction, on the timing of the emergence of these abilities.

## 2. Experimental Section

### 2.1. Participants

Participants were recruited from the subject pools of three labs in Munich (Max Planck Institute for Human Cognitive and Brain Sciences), London (Neurocognitive Development Unit, Institute of Child Health), and Paris (Developmental Neurocognition Unit, Laboratory of Cognition and Development). Parents were sent a brief letter introducing the study, together with a short preliminary questionnaire to gather some background data about the child and parents, to ask whether they wished to take part in this particular study and could commit to attending for one half day at 6 months of age and another half day four months later at 10 months of age. Infants were selected between 15 days prior and 15 days post six months, and between 15 and 20 days prior and post ten months. Table 1 provides detailed participant characteristics. Exclusion criteria were: prematurity (<37 weeks gestation), history of neurological problems previously diagnosed by a family doctor, atypical hearing or vision previously diagnosed by a professional, other languages spoken at home than the default language of each laboratory. Exclusion criteria were assessed through a parental questionnaire conducted during recruitment. A total of 122 infants were entered into the study at 6-months and 106 were retained for follow up at 10-months.

**Table 1:** Participant characteristics. Initial *n* reflects participants entered in the study at the time of recruitment. By 10 months some were lost to follow-up. A subset of these infants at each age produced sufficient valid trials to be included in the analysis. For those infants, the number of familiarization trials and looking time during familiarization is included.

	6 month		10 months	
	Native	Non-Native	Native	Non-Native
	M(SD)	M(SD)	M(SD)	M(SD)
Initial *n*	122		106	
Males:females	63:59		55:51	
*n* Remaining in the analysis	80	72	76	75
Number of familiarization trials (*sd*)	9.1 (5.1)	8.7 (5.3)	7.2 (4.4)	7.4 (4.3)
Looking time during familiarization (*sd*)	40.1 (26.7)	61.2 (20.4)	55.1 (26.8)	50.2 (20.6)

### 2.2. Methods

#### 2.2.1. Assessment of Speech Perception Skills

Testing took place in each of the three laboratories that had a similar set up. Infants were seated on their parent’s lap or on a special infant seat in front of a television monitor where the stimuli were presented. The setup was adjusted in each of the three labs so that the visual angle was the same. Plain curtains were drawn on each side of the infants to avoid interference from irrelevant stimuli. Speakers hidden behind black curtains to the left and right of the monitor delivered the speech sounds. The experimenter and a technician monitored the tasks in an adjacent room (or behind a curtain). 

*Stimuli:* English (or French and German) syllables were recorded at the London laboratory by trained native female speakers of each language. The English/French/German pairs—/ba/ *vs.* /da/—were chosen because they are distinctive in all three languages. Furthermore, this contrast has been previously used in a range of studies demonstrating the perceptual narrowing phenomenon [14]). Twenty exemplars of each sound were recorded by each native speaker. Final exemplars were chosen so that variations in duration, fundamental frequency and intonation contour were randomized both within and between phonetic categories. There were four tokens of the target syllable in the familiarization phase, and two tokens of the target, as well as the contrast syllables, in the test phase. 

The non-native contrasts were chosen from the Hindi language, which distinguishes four places of articulation (labial, dental, retroflex, and velar) in contrast to the three used in English, French and German (labial, alveolar, and velar). Dental stops are produced by obstructing air flow by placing the tongue back and at the top posterior to the alveolar ridge. The differentiated retroflex and dental Hindi stop consonants would *both* be typically categorized as alveolar- [t], by a naïve adult English, French, or German listener. The Hindi pairs were identical to those used in [14], allowing comparability and replicability of the effects in our experiment. These were minimal pairs of dental *vs.* retroflex contrasts (voiceless, not aspirated) that existed in none of the three native languages of the participants in the study. Detailed spectrograms and other characteristics of these Hindi stimuli can be found in the original study [14].

*Procedure:* Each infant was tested on a speech perception task in their native language (English, German, or French) and in Hindi. The order of administration of the two tasks was counterbalanced across infants. The native and non-native speech perception tasks were identical except for the speech categories used. A modified version of the familiarization-preference procedure [21] was used to assess discrimination. Testing was conducted in sessions lasting around five minutes each. In the familiarization phase, half of the infants were familiarized with [da] and the other half with [ba] until they reached criterion, that is, until they accumulated 1.5 min of sustained attention to this material. To begin, two sets of familiarization tokens from the same category were presented from the left and right loudspeakers. The maximum length for each trial was around 30 sec, which meant that infants needed at least three familiarization trials to reach criterion before proceeding to the test phase. Infants not meeting these criteria were not included in the analysis.

In the test phase, there were four test trials, two in which new tokens of the same category as the one in the familiarization phase were presented (same trials) and two in which new tokens of the contrastive category were heard (switch trials). The structure of the test trials was similar to the familiarization ones. The sequence of trial presentation in the test phase was quasi-random: the trial could begin with either a same or a switch trial, but no two same or two switch trials could be the first two trials of the test phase.

A trial started with an image on the center screen to capture the infant’s attention. As soon as s/he began to look at it, the image disappeared and a different one appeared on one of the two sides. When the infant looked in that direction, the presentation of the test stimuli began and continued until its completion or until the infant ceased to look for more than two consecutive seconds. Shorter non-looking times did not determine the end of the trial but were subtracted from the total listening time of that trial. Each infant was tested on all test trials. The side of presentation was randomized across trials. The video coding allowed us to determine listening time by measuring the length of the infant’s sustained attention to the dynamic stimulus paired with the speech contrast. 

All videos were coded off-line by trained coders blind to language category and test category, *i.e.*, coding was based on the infant’s orienting toward the stimulus being presented, without sound. The number of trials taken to reach criterion and length of looking time during the Familiarization and Test phases were calculated (detailed in Table 1). Inter-rater reliability calculated for 24 tests was 0.92 (Cohen’s Kappa).

#### 2.2.2. Assessment of Mother-child Interaction

The assessment procedure consisted of a 3 to 5-min videotaped mother-infant play interaction. The interaction was analyzed using the Care-Index [22], a validated manualized procedure assessing playful interaction occurring under non-threatening conditions. As per the manual instructions, adult-child dyads were video-taped for about 6 min in the lab, in a break between tasks. A blanket was spread on the floor and a standardized set of toys in all three labs was offered to the mother. The mother was instructed to play with her infant as she usually does. Although they were not compelled to play on the floor or use the toys, most mothers did so. The coders used the video segment after the dyad have settled into the interaction.

The Care-Index has been used in several studies including normative and clinical ones (e.g., [23,24]). Dyadic characteristics assessed in early infancy using this method are associated with attachment patterns emerging later in development. According to the manual, mother-infant dyads in our study were rated on a number of qualitative scales, some global and others describing specific behavioral aspects. The behavior of adult and child is assessed on seven dimensions: facial, vocal, position and body contact, affect, turn-taking contingencies, control, and choice of activity. The first four dimensions address various affective aspects, whereas the remaining dimensions address various aspects of the temporal contingency in the interaction. Of specific interest to our study is a sensitivity scale because it describes patterns of behavior that please the infant and increase his/her comfort and attentiveness and reduce distress and disengagement. The sensitivity rating, which ranges from 1–14 points, is derived based on coding of expressive channels in both the care-giver and the infant, according to the seven scales above. Each of the dimensions is assessed separately for the adult and the child in terms of the three adult scales (sensitive, controlling, unresponsive) and the four infant scales (cooperative, compulsive/compliant, difficult, passive). Two points are allocated to each dimension, yielding a total of 14 points. Although the scale provides separate ratings for mother and infant behavior, the scale does not assess these characteristics independently from one another. Hence, the scale provides a measure of the quality of dyadic interactions. An elevated score on this scale reflects highly contingent dyadic interactions, whereas a low score on this scale reflects asynchronous, less contingent dyadic interactions. 

The rating was performed by trained coders, who were independent from the coders who analyzed the speech perception task. Inter-rater reliability was established between two coders. Both coders first rated *n* = 10 participants independently. After discussing divergent cases and agreeing on changes, participants were re-coded. Reliability in the Munich lab was then *r* = 0.975 for the sensitivity scale, *r* = 0.974 for the control scale and *r* = 0.986 for the unresponsiveness scale. For the across-lab inter-rater reliability, two coders from each lab coded the same set of tapes. Twelve tapes were shared for reliability purposes between Munich and London and 21 tapes between Munich and Paris (approximately half from each lab). Scores were lower relative to within-lab reliability, since no subsequent attempt at obtaining mutual agreement was made. Between the Munich and the London lab reliability for on the sensitivity scale was *r* = 0.525, for the control scale was *r* = 0.461 and for the unresponsiveness scale was *r* = 0.946. Between the Munich and the Paris lab reliability on the sensitivity scale was *r* = 0.77, for the control scale *r* = 0.38, and for the unresponsiveness scale *r* = 0.84.

## 3. Results

**Figure 1:**
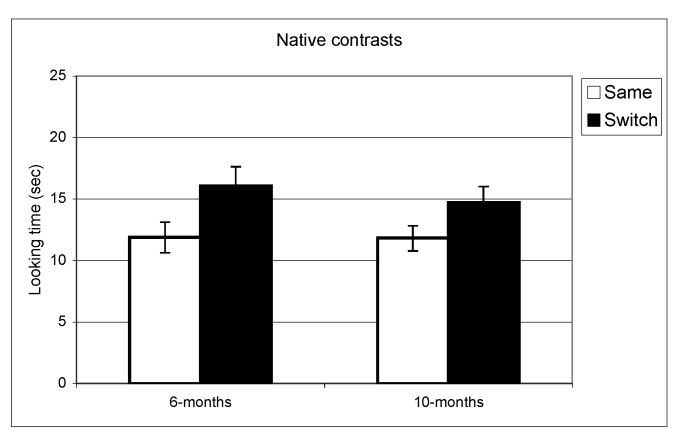
Looking time during same and switch trials for native contrasts at 6- and 10-months.

**Figure 2:**
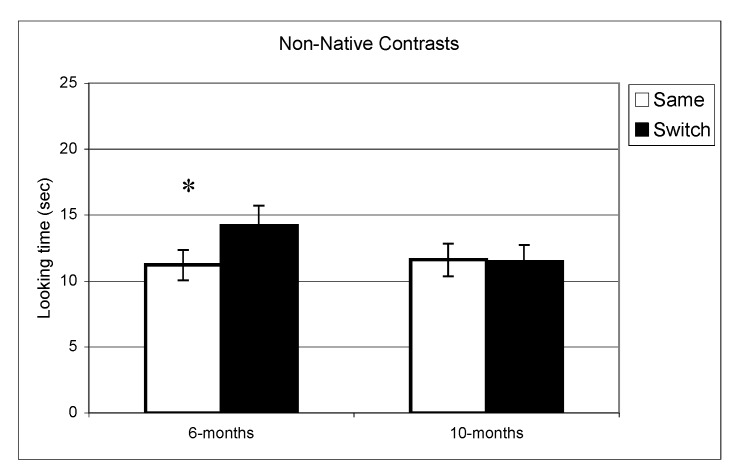
Looking time during same and switch trials for non-native contrasts at 6- and 10-months (* p < 0.05).

Table 1 shows the amount of data retained from the initial group of 122 infants at 6-months and 106 returning at 10-months follow up. Infants were excluded from the study due to fussiness and fatigue. Specifically, to be included in the analysis, infants had to complete all test trials either at 6-months, 10, months or both ages in either the native or non-native contrast. This approach allowed us to retain sufficient statistical power to examine the effects of subgroups based on parent-child interactions (see below). Figure 1 and Figure 2 show the amount of time infants attended to the syllables during same and switch trials at 6 and 10 months in the two experiments assessing perception of native and non-native speech contrasts. Differences in looking time between same and switch trials were analyzed using four ANOVAs corresponding to each experiment (Native *vs.* Non-Native) tested at baseline and again at follow-up. Each model included the factors Trial (same *vs.* switch) and lab membership (London, Munich, Paris) to verify that the target variable Trial was not affected by testing location. 

In the experiment assessing perception of native contrasts, results showed a significant main effect of Trial (same *vs.* switch), indicating that infants listened significantly longer during switch trails relative to same trials (*F*(1,77) = 19.5, *p* < 0.001). This pattern did not differ depending on the lab from which the data were collected; there was no interaction between Trial and Lab membership (*F*(2,77) = 0.65, *p* = 0.53). A similar pattern was observed at 10 months where there was a significant main effect of Trial (*F*(1,73) = 10.1, *p* = 0.002) but no interaction between Trial and Lab membership (*F*(2,73) < 1, *p* = 0.47). The same analysis was used for the non-native speech perception experiment. The results indicated a significant difference between same and switch trials on non-native contrasts at 6 months (*F*(1,69) = 8.9, *p* = 0.004) whereas at 10 months, no such difference in looking time emerged (F(1,72) < 1, *p* = 0.92). Also here, the interactions between Trial and Lab membership were not significant (both *p* > 0.2). In view of these findings, lab membership was dropped as a factor from subsequent analysis. 

In summary, these results indicate that as a group the 6-month-olds listened significantly longer during switch trials relative to same trials for both native and non-native contrasts. At 10-months, however, the discrimination effect was only found for native contrasts. To assess the relationship between the quality of mother-child interaction and speech discrimination performance, we split infants into two subgroups on the basis of the global rating on the sensitivity derived from the Care-Index. The overall mean for the sensitivity scale was 10 out of 14 (*SD *= 3.2) at 6 months and 11 out of 14 (*SD* = 2.9) at 10 months. Scores on maternal sensitivity were very strongly correlated (*r* = 0.8, *p* < 0.001) with another Care-Index measure describing infant cooperativeness, indicating that the scores reflected dyadic characteristics. Nevertheless, we preferred to use maternal scores to guarantee that any results obtained were not solely attributable to the general infant characteristics that might affect their speech discrimination performance. Dyads were divided into two groups whose scores fell above and below the median score on the sensitivity scale (11 at 6 and 10 months). Furthermore, although maternal sensitivity scores at 6-months and 10-months were very strongly correlated (*p* < 0.001), the group division was done separately for each age. Out of a maximum of 14, mean scores at both ages in the “high contingency” group were around 12 for maternal sensitivity and 12 for infant cooperativeness, whereas mean scores in the “moderate contingency” group were around 7 for maternal sensitivity and 8 for infant cooperativeness. 

**Figure 3:**
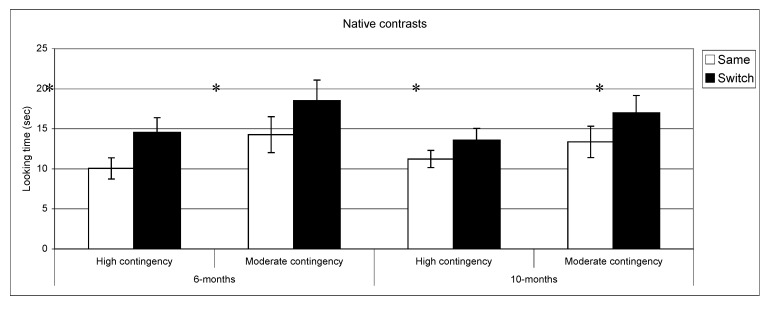
Looking time during same and switch trials for native contrasts at 6- and 10-months for subgroups of infants (* *p* < 0.05).

**Figure 4:**
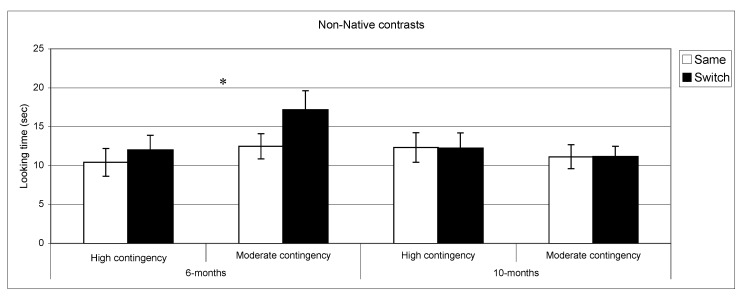
Looking time during same and switch trials for non-native contrasts at 6- and 10-months for subgroups of infants (* *p* < 0.05).

Figure 3, Figure 4 present discrimination results for native and non-native contrast as a function of contingency of interaction. Post-hoc comparisons (setting a *p* value < 0.01 to correct for multiple comparisons) confirmed longer looking in switch *vs.* same trials for native phonemes across the two groups at both ages: high contingency 6 months, *t*(39) = −3.9, *p* < 0.001, *n *= 40; high contingency 10 months, *t*(49) = −2.2, *p* = 0.02, *n *= 50; moderate contingency 6 months, *t*(36) = −2.6, *p* = 0.01, *n *= 37; moderate contingency, 10 months *t*(28) = −2.3 *p* = 0.02, *n *= 29. By contrast, in the non-native speech perception tasks, the results of the subgroups differed from the overall group results. At 6 months, infants in the high contingency group did not show a significant difference between same and switch trials (*t *(32) = −1.02, *p* = 0.31, *n *= 33), whereas the infants in the moderate contingency group displayed this difference (*t *(34) = −3.1, *p* = 0.004, *n *= 35). At 10 months, performance in both groups parallels the overall group results where infants in both groups showed no differences in listening time for same *vs.* switch trials. 

To ascertain whether these differing results in the High and Moderate contingency groups were associated with differences in attention to the stimuli during the task, we assessed whether the groups differed in the amount of sustained attention to the stimuli in the non-native discrimination experiment at 6 months. The results indicated that total looking time in the familiarization phase was remarkably similar (high contingency = 61.4, *SD* =17.4; moderate contingency =61.9; *SD*=24.1, *p *= 0.9). Hence, the results cannot be attributed to differences in exposure time during the familiarization phase.

## 4. Discussion

The relationship between phonetic development and the specific context of language acquisition, has rarely been investigated in previous research [19]. Our findings suggest that specific characteristics of this social context, measured in our study through differences in the quality of dyadic interactions, exert an influence on the timing of this developmental process. At 6 months, infants from dyads with high contingency scores appear to have already narrowed their perceptual abilities to their mother tongue, yielding evidence for early specialization in that they discriminated native but not non-native speech contrasts, whereas infants from dyads with moderate contingency scores continue to discriminate both native and non-native contrasts. By 10 months of age, the two groups of infants were indistinguishable, both displaying the expected pattern of discrimination for their native but not for the non-native phonetic categories. 

Our findings suggest that characteristics of the social environment maps onto the timing of native language discrimination. This developmental milestone has already been viewed as critical in setting the stage for other linguistic skills acquired well into toddlerhood. The timing of this ability at around 10 to 12 months of age coincides with the point at which children begin to understand meaningful words and to produce the particular sounds of their native language [3]. Some have suggested that these evolving perceptual skills are essential for segmenting the speech stream and for mapping words onto meaningful concepts, in a process where phonological and lexical acquisition go hand in hand [25,26]. Longitudinal studies have revealed that perceptual sensitivity at 6 months predicts vocabulary size at 24 months [27] as well as reading skills at 3–8 years of age [19,28]. 

What are the implications of these results for theories of language acquisition? Traditionally, the debate over language acquisition in general, and phonetic development in particular, has focused on whether the infant’s evolving capacities are subserved by “modular”, domain-specific mechanisms or, alternatively, by general perceptual mechanisms [29,30]. In view of our findings, the relationship between mother-child interactions and phonetic discrimination performance does not appear to be mediated by a general mechanism, e.g., categorization skills, since the effects seen were specific to non-native, but not to native language contrasts. Instead, our findings support the emerging view of language acquisition as drawing on a diverse set of perceptual, cognitive, and social mechanisms [19,31]. The developmental relations among these mechanisms and phonetic development can be captured within specific time windows, in our study at 6 months but not at 10 months, where individual variability in the timing of phonetic specialization is influenced by the social context of language acquisition. Hence, while phonetic development initially draws on broad perceptual mechanisms, further specialization and in particular its timing is influenced by a variety of mechanisms including social ones. 

What might be driving these differences in speech discrimination performance as a function of the quality of mother-child interaction? One possibility is that mothers in the high contingency group provide more linguistic input to their infants compared to the moderate contingency group. This, however, is inconsistent with previous studies demonstrating that mere exposure to linguistic contrasts outside the social context is not sufficient for successful discrimination [19]. Furthermore, while previous research has highlighted the critical role of statistical learning in lexical segmentation, it has also affirmed that human infants are efficient learners [13]. Consistent with this view, in the ratings of mother-child interactions in the instruments we employed, frequency of input was not a primary factor in discrimination. On the other hand, it has been previously suggested that the social context aids learning through eliciting attention and motivation and this even extends to other species such as birds in learning songs [19]. Our findings offer further insights into the features that characterize these interactions and how they might facilitate learning through stimulating the infant’s attention and motivation. Dyads in the high contingency group showed more mutual gaze, verbal and non-verbal turn-taking, and mutual affect. Furthermore, these interactions were characterized by high levels of contingency, where mothers altered their behavior as a function of the infants’ behavior and varied their verbal and non-verbal input within the context of these contingent interactions. Taken together, our findings are consistent with previous work suggesting that quantity of input is only one among many factors that need to be accounted for within the social context of language acquisition. 

Further support for the importance of dyadic contingency in relation to phonetic learning comes from atypical development. In the neurodevelopmental disorder autism, differentiation of native language phonetic categories is less clear than that found in typical development, even by the age of 3–4 years [32]. While a number of studies have shown that mothers of children with autism do not differ in the frequency of verbal input they provide, the interactions are less synchronous than those seen in typically developing infants and their care-givers [32]. These atypical patterns of social interactions appear to result in serious consequences for phonetic development and, as our current study has shown, even in the typical case, features of mother-child interaction influence the timing of infants’ specialization for the sounds of their native language. 

Our findings require replication because of two key limitations. First, despite the large sample size we did not have sufficient statistical power to track each infant’s development longitudinally across native and non-native phoneme discrimination tasks. Given typical limitation of infancy experimental research designs, only a handful of infants produced valid data across both ages and in both experiments. Therefore, future studies need to have larger samples or employ novel methods to reduce data attrition. The second limitation of our study is that we did not specifically explore potential interactions between the specific language environment (English *vs.* French *vs.* German), parental characteristics such as education and socioeconomic status, and patterns of mother-child interaction. Therefore, such potential cross-linguistic and individual differences need to be explored in more detail in future studies. 

## 5. Conclusions

Parent-infants dyads with high contingency in interactions showed early specialization for their native phonemes by failing to discriminate non-native contrasts. Our findings suggest that the quality of mother-child interaction is one of the exogenous factors influencing the timing of infant specialization for speech processing. Our findings reinforce the possibility that the development of the infant’s phonological system into the specialized adult categories, is subject to environmental influences. 
